# Yiguanjian decoction and its ingredients inhibit angiogenesis in carbon tetrachloride-induced cirrhosis mice

**DOI:** 10.1186/s12906-015-0862-6

**Published:** 2015-10-01

**Authors:** Ya-Ning Zhou, Yong-Ping Mu, Wen-Wei Fu, Bing-Bing Ning, Guang-Li Du, Jia-Mei Chen, Ming-Yu Sun, Hua Zhang, Yi-Yang Hu, Cheng-Hai Liu, Lie-Ming Xu, Ping Liu

**Affiliations:** Institute of Liver Diseases, Shuguang Hospital, Shanghai University of Traditional Chinese Medicine, Shanghai, 201203 China; Shanghai University of Traditional Chinese Medicine, Shanghai, 201203 China; E-institute of Shanghai Municipal Education Commission, Shanghai, 201203 China

**Keywords:** Cirrhosis, Angiogenesis, Yiguanjian

## Abstract

**Background:**

Cirrhosis is associated with angiogenesis and disruption of hepatic vascular architecture. Yiguanjian (YGJ) decoction, a prescription from traditional Chinese medicine, is widely used for treating liver diseases. We studied whether YGJ or its ingredients (iYGJ) had an anti-angiogenic effect and explored possible mechanisms underlying this process.

**Methods:**

Cirrhosis was induced with carbon tetrachloride (CCl_4_) (ip) in C57BL/6 mice for 6 weeks. From week 4 to week 6, cirrhotic mice were randomly divided into four groups: sorafenib-treated, YGJ-treated and iYGJ-treated mice and placebo. Serum biochemistries, hydroxyproline (Hyp) content and histopathological changes of hepatic tissues were measured as were α-smooth muscle actin (α-SMA), collagen I, CD31, vascular endothelial growth factor (VEGF), VEGF receptor (VEGFR) 2 and hypoxia-inducible factor (HIF)-1α.

**Results:**

Both YGJ and iYGJ improved serum biochemistries. Changes of histopathology showed that YGJ and iYGJ reduced hepatic tissue necroinflammatory and collagen fiber deposition in cirrhosis mice. Compared to the CCl_4_ treated animals, Hyp, α-SMA, collagen I, CD31, VEGF, VEGFR, and HIF-1α expression decreased in YGJ and iYGJ groups.

**Conclusions:**

YGJ and iYGJ inhibited liver angiogenesis in cirrhotic mice treated with CCl_4_ by inhibiting the HIF-1α/VEGF signaling pathway, suggesting that anti-angiogenic effects of YGJ and iYGJ are associated with improving the hepatic hypoxic microenvironment.

## Background

One theory underlying the pathogenesis of cirrhosis is angiogenesis and disruption of hepatic vascular architecture. The increased vascular density and abnormal angio-architecture not only increase intrahepatic vascular resistance and decrease hepatocyte perfusion, but also modulate the formation of portal-systemic collaterals, which are responsible for cirrhotic complications such as portal hypertension [1]. Anti-angiogenesis has been a therapeutic strategy for cirrhosis and cirrhotic angiogenesis is regulated through two main pathways: overexpression of grow factors and cytokines, especially platelet derived growth factor (PDGF), transforming growth factor (TGF)-β1, fibroblast growth factor (FGF) and vascular endothelial growth factor (VEGF) in the process of hepatic chronic wound healing. Hypoxia contributes to up-regulation of the hepatic angiogenesis signaling pathway [2]. Angiogenesis is characterized by hypoxic stimulation and growth factor dependence. VEGF is the only specific mitogen for endothelial cells that undergoes mitosis and creates vascular tubes, stimulating proliferation and migration of endothelial cells, and thereby mediating physiological and pathological angiogenesis [3]. VEGF is constitutively expressed in endothelial cells and hepatocytes at low levels under normal physiological conditions. With hypoxia, hypoxia-inducible factor (HIF)-1α induces expression of downstream target genes, including VEGF and VEGF receptor (VEGFR) 2. Furthermore, hypoxia enhances VEGF mRNA stability to prevent degradation and to increase transcription of VEGF [4].

Yiguanjian (YGJ) decoction was first described in ancient traditional Chinese medicine and has been used to treat hepatic diseases in China for centuries. YGJ decoction has 6 constituents: *glehniae radix, ophiopogonis radix, angelicae sinensis radix, dried rehmanniae radix, lycii fructus and toosendan fructus*. Research indicates that hepatoprotective and anti-fibrogenic effects of YGJ against dimethylnitrosamine (DMN)-induced hepatic injury. In DMN-induced rats, oral administration of YGJ significantly reduced the serum aspartate aminotransferase (AST) and alanine transaminase (ALT), and inhibited accumulation of collagen I, tissue inhibitor of metalloproteinase-1, and α-smooth muscle actin (α-SMA) in hepatic tissues [5]. Recent research indicates that YGJ improved liver fibrosis by inhibiting the migration of bone marrow cells into the liver, inhibiting their differentiation and suppressing the proliferation of both progenitor cells and hepatocytes in injured livers [6]. We previously reported that YGJ exerts therapeutic effects on carbon tetrachloride (CCl_4_)-induced cirrhosis in rats, by inhibiting hepatocyte apoptosis and hepatic stellate cell (HSC) activation, as well as regulating the function of Kupffer cells [7]. In addition, modified YGJ induced HSC apoptosis via reactive oxygen species accumulation and inducing the intrinsic apoptotic pathway *in vitro* [8]. Another modified YGJ induced hepatocarcinoma cell anoikis *in vitro*, which may be associated with down-regulation of p38 MAPK [9]. In our previous study, YGJ was extracted using five different methods and we identified several active ingredients (iYGJ) that may suppress liver fibrosis. iYGJ improved hepatic function and reduced extracellular matrix (ECM) accumulation in CCl_4_-induced cirrhosis in mice (data not published). However, how YGJ and iYGJ prevents angiogenesis is uncertain. Thus, we investigated the effects of YGJ and iYGJ on angiogenesis and studied the mechanisms underlying this process.

## Methods

### Materials

CCl_4_ was purchased from Guoyao Company (China). Hydroxyproline (Hyp) standard was purchased from Nakateitesuku Corporation (Japan). Biochemical parameters kits were purchased from Nanjing Jiancheng Biotech Company (China). Protein assay reagent was purchased from Thermo Scientific (USA). Antibodies against α-SMA, collagen I, CD31, VEGF and VEGFR2 were obtained from Abcam (UK). Antibody against HIF-1α was obtained from Biotechnology CO.,Ltd (China). Second antibodies were obtained from LI-COR (USA). The RNA isolation kit, reverse transcriptase kit and SYBR Green PCR Master Mix were obtained from Takara (Japan). Sorafenib was used as positive control in this experiment and was manufactured by Bayer Pharmaceuticals (West Haven). Verbascoside and ferulic acid were purchased from Chengdu Must Biological Technology CO.,Ltd (China). Compound purities were greater than 98.0 % as confirmed by high-performance liquid chromatography (HPLC).

### Preparation of YGJ and iYGJ

Six herbs for the study were purchased from Shanghai Huayu Chinese Herbs Co.,Ltd. (Shanghai, China), *glehniae radix* (lot #2010092417); *ophiopogonis radix* (lot #110307); *angelicae sinensis radix* (lot #2011020917); *dried rehmanniae radix* (lot #2011010606); *lycii fructus* (lot #2011032802); *toosendan fructus* (lot #2011020040). All of them were identified by Dr. Wang of Shanghai University of Traditional Chinese Medicine. A voucher specimen of each species was deposited at the Herbarium of the School of Pharmacy, Shanghai University of Traditional Chinese Medicine. YGJ was prepared by the department of pharmaceutics, Shuguang hospital. Briefly, YGJ herbs, including *glehniae radix* 10 g, *ophiopogonis radix* 10 g, *angelicae sinensis radix* 10 g, *dried rehmanniae radix* 18 g, *lycii fructus* 12 g and *toosendan fructus* 4.5 g, were boiled under reflux three times. The filtrate was concentrated and dried to powder, and 1 g of the extract contained 2.3 g herbs.

*Extraction of iYGJ*. The aqueous extract prepared as above was mixed with three times volume of 95 % ethanol and then kept overnight in a refrigerator. The supernatant was evaporated *in vacuo* to remove, and then the aqueous extract was chromatographed over a macroporous resin D101 column and eluted with H_2_O, 30 %, 60 % and 95 % EtOH. The 30 % EtOH elution was concentrated and dried to powder and 1 g of the extract contained 47.1 g herbs.

### Analysis on the fingerprint of iYGJ by HPLC

**Fig. 1:**
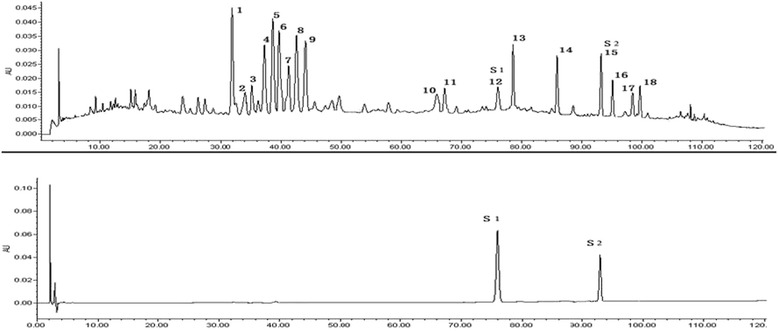
Main HPLC fingerprinting of iYGJ decoction. S1: verbascoside; S2: ferulic acid

The analysis of the iYGJ fingerprint was performed on a Waters 2695 HPLC system equipped with a vacuum degasser, a quaternary pump, an autosampler and a Waters 2487 UV detector system, connected to Waters Empower software. A Diamonsil C_18_ column (250 mm × 4.6 mm, 5 μm) was used for separation. Optimum separation was achieved using gradient elution with 0.5 % formic acid in water (A) and methanol (B): 0–10 min (5 %B–10 %B), 10–60 min (10 %B–20 %B), 60–100 min (20 %B–40 %B), 100–120 min (40–90 %B), 120–130 min (90 %B). The flow rate was set at 1.0 ml/min and the injection volume was 20 μl. Column temperature was maintained at 30 and UV detection was read at 330 nm. The HPLC fingerprint of iYGJ is illustrated in Fig. 1.

### Animal model

C57BL/6 male mice, SPF, 20 ± 2 g, are supplied by the Shanghai Experimental Animal Center, Chinese Academy of Sciences. Animals were maintained on standard chow in an air-conditioned room with a 12 h dark/light cycle. All study protocols were approved by the Animal Ethics Committee of Shanghai University of Traditional Chinese Medicine.

Cirrhosis was induced by 10 % CCl_4_ (2 ml/kg, ip) diluted with olive oil, 3 times a week for 6 weeks. Controls (*n* = 10) were given olive oil only. From week 4 to week 6, CCl_4_-injected mice were divided into four groups (*n* = 10 each group except for the placebo group *n* = 15) and then subsequently treated with sorafenib (4 mg/kg), YGJ (4 g/kg), iYGJ (0.2 g/kg) and placebo (water) respectively by gavages, once a day. The controls were given water. In the same time, CCl_4_ or olive oil was injected respectively.

All mice were sacrificed after pentobarbital anesthesia. Blood samples were obtained from interior vena cava, centrifuged at 3,000 rpm for 15 min, and serum was kept for serum biochemistry tests. Liver tissue specimens were taken from the right hepatic lobe and fixed in 10 % phosphate-buffered formaldehyde, routinely processed, and then embedded in paraffin. Additional liver tissues were snap-frozen in liquid nitrogen and stored at -70 °C for later analysis.

### Serum biochemistry and Hyp assay

Serum biochemistries—ALT, AST and albumin (ALB)—were measured as indicated by their assay standard kits. Hyp assay of liver tissue was conducted on a modified version of a method previously described [10]. Liver tissue was homogenized and hydrolyzed in 6 N HCl at 110 °C for 18 h. The hydrolysate was filtered and to this chloramine-T was added (final concentration 2.5 mM). The mixture was then treated with 410 mM paradigm ethyl-amino-benzaldehyde and incubated for 30 min at 60 ^o^C. Finally samples were read at λ_560 nm_.

### Hepatic histopathology and immunohistochemistry

Hepatic histopathology and immunohistochemistry were assessed by staining paraffinized liver sections (4 μm) with hematoxylin and eosin (H&E), and Sirius red. For immunohistochemistry, sections was incubated with primary antibody (α-SMA 1:200, collagen I 1:250, CD31 1:200, and VEGF 1:200) overnight, and then incubated with secondary labeled polymer HRP antibody for 1 h followed by DAB staining. Collagen I was semi-quantified using image analysis software. Five fields of each section were randomly selected and values are expressed as means of five animals per group.

### Western blot

Liver samples were homogenized for 10 s at 10,000 rpm 3 times. Total protein was measured using a protein assay reagent, and 50 μg protein was separated by SDS-PAGE and then electrophoretically transferred to nitrocellulose membranes. After incubation in blocking buffer, the membrane was incubated with primary antibody (α-SMA 1:1,000, CD31 1:1,000, VEGF 1:1,000, VEGFR2 1:1,000, and HIF-1α 1:1,000) at 4 °C overnight, followed by incubation with secondary antibody at room temperature for 1 h. Immunoblots were visualized with an infrared imaging system (Odyssey). Finally, the membrane subsequently was treated with anti-GAPDH antibody.

### RNA isolation and RT-PCR

Total RNA was extracted from snap-frozen liver tissue samples according to kit instruction. Total RNA was reverse transcribed using reverse transcriptase kit according to the manufacturer’s guidelines. Primers for real time reverse transcription PCR (RT-PCR) were designed and synthesized by Takara (Japan). Primers used for RT-PCR are as follows:

VEGF forward primer, 5′-GAAAGGGTCAAAAAACGAAAGCG-3′,

VEGF reverse primer, 5′-TCTGCGGATCTTGGACAAACAA-3′,

VEGFR2 forward primer, 5′-TGCCTACCTCACCTGTTTCC-3′,

VEGFR2 reverse primer, 5′-CTCTTTCGCTTACTGTTCTGGAG-3′,

HIF-1α forward primer, 5′-GGACGATGAACATCAAGTCAGCA-3′

HIF-1α reverse primer, 5′-AGGAATGGGTTCACAAATCAGCA-3′

GAPDH forward primer, 5′-CTTTGGCATTGTGGAAGGGCTC-3′,

GAPDH reverse primer, 5′-GCAGGGATGATGTTCTGGGCAG-3′,

GAPDH expression was used as an internal control. Initial denaturation was performed at 95 °C for 30 s, followed by 40 cycles of 95 ^o^C denaturing for 5 s, and extension at 60 °C for 30 s. SYBR Green fluorescent intensity of specific double-strand reflecting the amplicon amount was read after each elongation step at an additional acquisition temperature. To verify specificity of the amplification reaction, melting curve analysis was performed. Relative gene expression was presented using the ΔΔCT method. RT-PCR was carried out using the ABI ViiA7 sequence detector (Applied Biosystems).

### Statistical analysis

All data were expressed as means ± SD. Statistical analysis was performed with SPSS software, version 17.0. Data were analyzed using homogeneity of variances and one-way analysis of variance (ANOVA). *P*-values less than 0.05 were considered statistically significant.

The animal model was from another study from which controls were shared. Data for ALT, AST, ALB, Hyp and collagen I area were shared [11].

## Results

### YGJ and iYGJ against CCl_4_-induced cirrhosis

At the end of the 6th week, serum ALT and AST activity were significantly higher in the CCl_4_ model group than in controls (*P* < 0.05). Sorafenib, YGJ and iYGJ reduced serum ALT and AST activity. Moreover, YGJ and iYGJ reversed decreased ALB induced by CCl_4_ (*P* < 0.05) (Table 1).

**Table 1:** Serum ALT, AST and ALB in each group (means ± SD)

Group	*n*	ALT(IU/L)	AST(IU/L)	ALB(g/L)
Control	10	22.3 ± 2.9	31.4 ± 4.8	32.4 ± 2.2
CCl_4_	15	121.7 ± 30.0 ^#^	67.6 ± 14.6 ^#^	29.3 ± 1.8 ^#^
CCl_4_ + Sorafenib	10	73.8 ± 16.7^#^*	52.6 ± 8.7^#^*	31.6 ± 1.2
CCl_4_ + YGJ	10	77.9 ± 14.5^#^*	52.8 ± 9.5^#^*	32.6 ± 3.2*
CCl_4_ + iYGJ	10	72.6 ± 12.8^#^*	44.5 ± 8.8^#^*	32.7 ± 1.3*

Note: #, compared to control group *P* < 0.05; *, compared to CCl_4_ group, *P* < 0.05

**Fig. 2:**
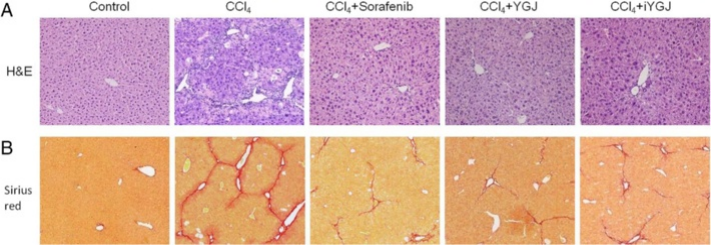
Effects of YGJ and iYGJ on CCl_4_-induced liver injury. Histopathology was assessed with H&E (**a**, 200×) and Sirius red (**b**, 100×)

H&E staining revealed hepatocytes arranged in cords from central veins to portal area, with intact hepatic lobules in normal control mice. Damaged lobules and extensive necroinflammatory lesions were visible in CCl_4_ group livers. Compared to the CCl_4_ group, necroinflammatory hepatic lesions were reduced in groups treated with sorafenib, YGJ and iYGJ (Fig. 2a). Sirius red staining confirmed that modest collagen was present in the area of the portal and central veins in normal control mice, but in the CCl_4_ group, bridging collagen connecting the central veins and neighboring portal areas increased, and collagen fiber deposition was increased and pseudonodules were present. In contrast, in the sorafenib, YGJ and iYGJ groups, hepatic collagen deposition were significantly attenuated (Fig. 2b).

Consistently, hepatic Hyp content was significantly increased after CCl_4_ treatment compared with controls. However, sorafenib, YGJ or iYGJ treatment decreased Hyp content compared with CCl_4_ treated animals (*P* < 0.05; Table 2).

**Table 2:** Hyp content in each group (means ± SD)

Group	*n*	Hyp (μg/g)
Control	10	133.0 ± 31.3
CCl_4_	15	411.2 ± 67.6^#^
CCl_4_ + Sorafenib	10	276.7 ± 30.8^#^*
CCl_4_ + YGJ	10	240.5 ± 45.4^#^*
CCl_4_ + iYGJ	10	254.5 ± 29.9^#^*

Note: #, compared to control group *P* < 0.05; *, compared to CCl_4_ group, *P* < 0.05

**Fig. 3:**
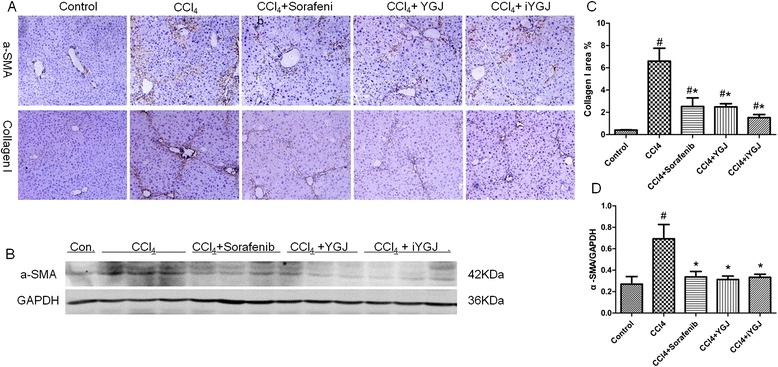
Effects of YGJ and iYGJ on CCl_4_-induced activation of HSC. **a** Immunohistochemistry of liver sections for α-SMA and collagen I (200×). **b** Intensity of collagen I (A, below) was assessed by image analysis. *n* = 5. **c** Western blot quantified protein expression of α-SMA. GAPDH expression was a control for equal protein loading. **d** Quantification of band intensities of expressed proteins. *n* = 3. Quantitative data were reported as means ± SD. #, compared to control group *P* < 0.05; *, compared to CCl_4_ model group *P* < 0.05

Immunohistochemistry indicated that accumulation of the active HSC marker α-SMA in livers increased in the CCl_4_ group. This change was consistent with protein expression of α-SMA analyzed by Western blot, which also increased after CCl_4_ injection. Increased expression of α-SMA was followed by enhanced collagen I synthesis. Semi-quantification of immunohistochemical staining revealed that collagen I increased sharply after CCl_4_ injection, 6.5-fold above control values. After treatment with sorafenib, YGJ or iYGJ, expression of α-SMA decreased according to immunohistochemistry data, and this agreed with data confirmed by Western blot. Thus, collagen I expression was significantly suppressed by sorafenib, YGJ or iYGJ treatment (*P* < 0.05; Fig. 3).

### Effects of YGJ and iYGJ on angiogenesis

**Fig. 4:**
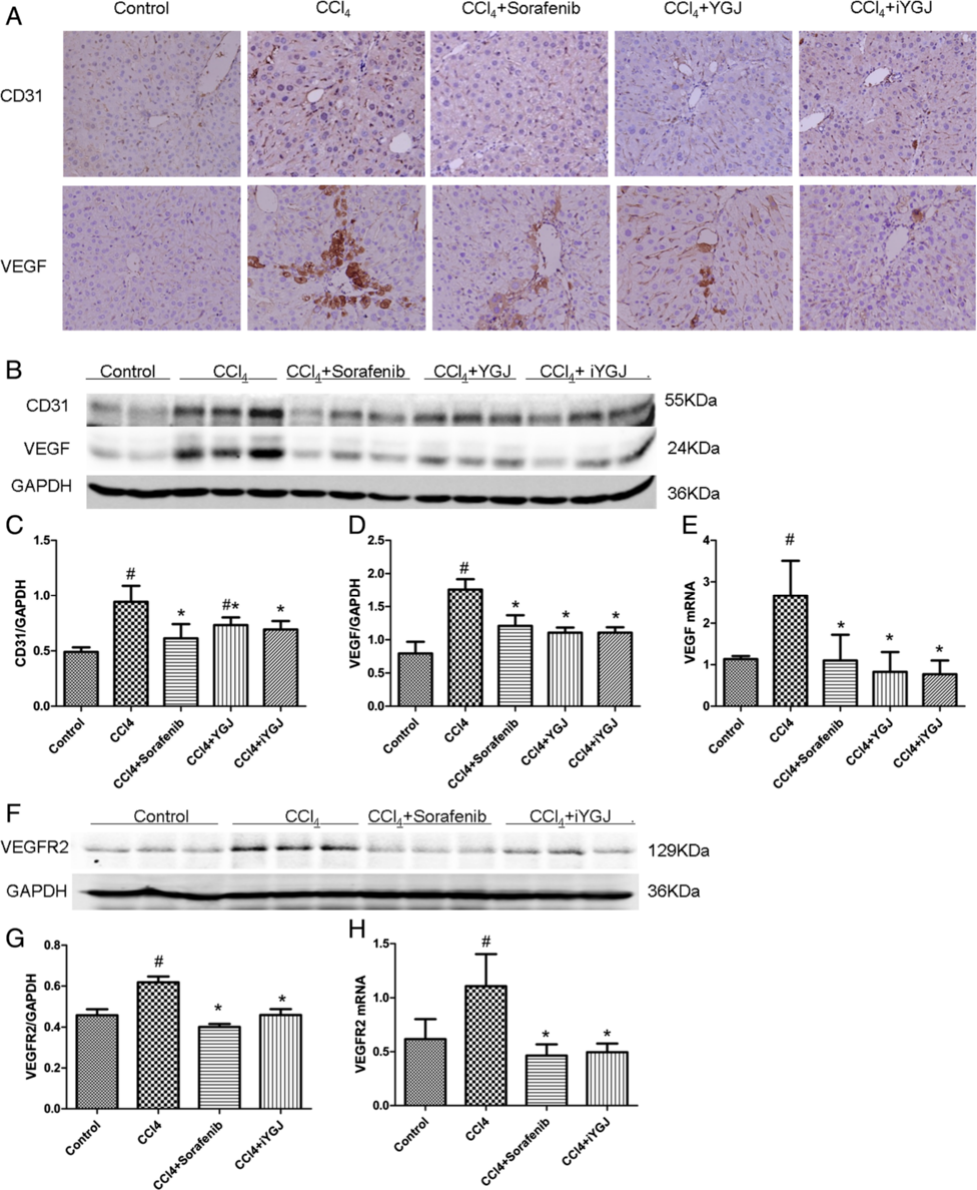
Effects of YGJ and iYGJ on CCl_4_-induced liver angiogenesis. **a** Immunohistochemistry of liver sections for CD31 and VEGF (400×). **b**, **f** Western blot quantified protein expression of CD31, VEGF and VEGFR2. GAPDH expression was a control for equal protein loading. **c**, **d**, **g** Quantification of band intensities of expressed proteins. *n* = 3. **e**, **h** VEGF and VEGFR2 mRNA expression was measured by RT-PCR. *n* = 3. Quantitative data were reported as means ± SD. #, compared to control group *P* < 0.05; *, compared to CCl_4_ model group *P* < 0.05

Immunohistochemistry revealed that CD31 and VEGF expression in the liver was up regulated after CCl_4_ treatment compared to controls. Notably, VEGF was observed not only in the hepatic sinusoid, but also in injured hepatocytes adjacent to the fibrotic septa around the portal area. In contrast, positive staining for CD31 and VEGF were decreased in the YGJ, iYGJ and sorafenib groups (Fig. 4a). Data from Western blot indicate that sorafenib, YGJ or iYGJ treatment reduced over-expression of CD31 and VEGF induced by CCl_4_, which is consistent with results of immunohistochemistry (*P* < 0.05; Fig. 4b, c, d). Moreover, VEGF mRNA was significantly increased in the CCl_4_ group compared to controls, but after sorafenib, YGJ or iYGJ treatment expression significantly decreased compared CCl_4_ treatment (*P* < 0.05; Fig. 4e). We next measured the effect of iYGJ on VEGFR2 with Western blot and PCR and noted that after CCl_4_ treatment, VEGFR2 expression was up regulated, but in the iYGJ treated group, VEGFR2 decreased markedly compared to the CCl_4_ group (*P* < 0.05; Fig. 4f, g, h).

**Fig. 5:**
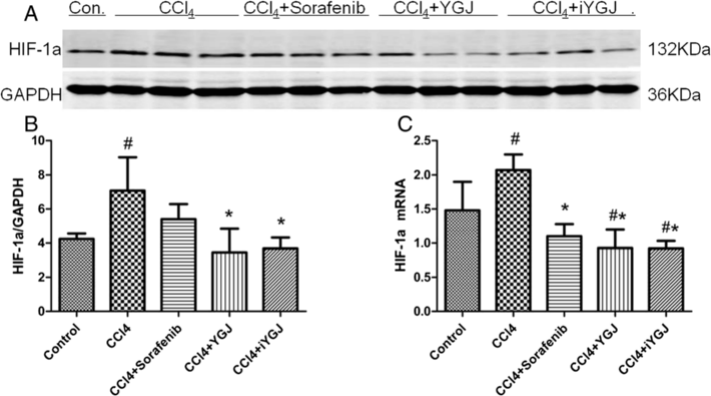
Effects of YGJ and iYGJ on HIF-1α. **a** Western blot quantified protein expression of HIF-1α. GAPDH expression was a control for equal protein loading. **b** Quantification of band intensities of expressed proteins. *n* = 3. **c** HIF-1α mRNA expression was measured by RT-PCR. *n* = 3. Quantitative data were reported as means ± SD, #, compared to control group *P* < 0.05; *, compared to CCl_4_ model group *P* < 0.05

Western blot and RT-PCR combined to indicate that expression of HIF-1α was significantly increased in the CCl_4_ group compared controls. As expected, protein and gene expressions of HIF-1α were all down regulated significantly by YGJ and iYGJ (*P* < 0.05). Also, the extent of YGJ and iYGJ in HIF-1α protein down-regulation was greater than after sorafenib treatment (Fig. 5).

## Discussion

Angiogenesis is a complex biological process in which new capillaries form, initiated from vascular sprouts based on pre-existing vascular construction. Neo-angiogenesis is an important pathophysiological feature of liver scarring, as evidenced by the presence of newly created blood vessels in fibrous septa around the portal area [1]. Many studies suggest that the multiple tyrosine kinase inhibitor (e.g. sorafenib and sunitinib) may have antifibrotic effects [12], but it cannot easily be applied for human liver diseases due to serious side-effects. Angiogenesis signaling is involved in the wound healing response in hepatic fibrosis, contributing not only to ECM deposition, but also to portal hypertension [2]. Classic angiogenenic mediators, notably VEGF and PDGF, drive both angiogenic and fibrogenic responses, and may also foster a milieu permissive for the development of hepatocellular carcinoma [13]. Therefore, safe and effective therapies targeting key molecules involved in anti-angiogenenic progression are urgently needed to prevent hepatic fibrosis.

Here, we show that YGJ and iYGJ effectively ameliorated liver injury stimulated by CCl_4_ in mice, improved liver function, decreased inflammatory infiltration and liver necrosis, decreased ECM deposition and decreased Hyp. Moreover, α-SMA and collagen I expression (secreted mainly by HSC) were inhibited by YGJ and iYGJ. Thus, antifibrotic effects of YGJ and iYGJ are partially mediated by inhibiting activation of HSC in CCl_4_-induced cirrhotic mice.

Hepatic sinusoids are lined by fenestrated endothelial cells which lack a basement membrane, and intercellular junctions are absent. This anatomy facilitates rapid exchange between plasma and hepatocytes. Morphologically, cirrhosis may be accompanied by widening of the space of Disse, subendothelial deposition of collagen and basement membrane material. These changes are often referred to as “capillarization of sinusoids”. Structural change reduces transport across the sinusoidal walls, leading to functional impairment. CD31 is identified as a marker generally used for labeling continuous endothelial cells, which can be expressed by the newly formed hepatic vasculature. Accordingly, the content and distribution of CD31 could reflect the condition of angiogenesis. In our study, immunohistochemistry and Western blot confirmed CD31 expression was increased in the liver of cirrhotic mice induced by CCl_4_, indicating that angiogenesis is a process of cirrhosis. After administration of YGJ or iYGJ, CD31 expression was decreased significantly, and these effects were similar to those conveyed by sorafenib (positive control). Sorafenib, a kinase inhibitor that targets multiple tyrosine kinases, is confirmed modulate angiogenesis and fibrosis. Thus, YGJ and iYGJ have the potential to inhibit angiogenesis of cirrhosis.

Hepatic angiogenesis is strongly linked to progressive fibrogenesis, although mechanisms of direct interactions between both processes are not yet entirely clear. Scar development and disorganization of the vasculature in cirrhosis may aggravate local hypoxia. Recently, hepatic disturbances in microcirculation, hypoxia, and angiogenesis have been documented to occur well before the onset of cirrhotic lesions in injured livers [14]. HIF-1, a heterodimeric comprised of a labile alpha subunit (HIF-1α) and a stable beta subunit (HIF-1β), is a major transcription factor for regulating oxygen homeostasis. The alpha subunit is regulated strictly by the concentration of oxygen, whereas the beta subunit is expressed in numerous cell types, and is not influenced by oxygen tension. Normally, the half-life of HIF-1α is very short. It is synthesized and degraded dynamically. When the concentration of oxygen is lowered (≤2 %), oxygen-dependent degradation of HIF-1α is inhibited [15]. The excessive accumulation of HIF-1α would induce transcription of downstream genes, including VEGF, PDGF and FGF [4, 14]. In particular, 70–80 % expression of VEGF mRNA depends on HIF-1α [16]. Our data indicate that YGJ and iYGJ significantly reduced HIF-1α and its downstream target gene, VEGF, at the protein and mRNA levels. Biological activity of VEGF is mediated mainly via VEGFR1 and VEGFR2. In the CCl_4_ model, vessel formation was associated with strong expression of VEGF and its receptor, VEGFR2 [17]. We report that iYGJ significantly inhibited expression of VEGFR2 in a CCl_4_ model. This indicates that the anti-angiogenic effect of YGJ and iYGJ was like caused by inhibiting the HIF-1α/VEGF signaling pathway. In addition, cirrhosis experimental model studies and analysis of patients with cirrhosis induced by hepatitis B virus infection indicate that hepatic VEGF expression mainly focuses on expansive sinusoid endothelial cells and severely injured hepatocytes [18–20]. In this current study, VEGF was observed in the cytoplasm of injured hepatocytes adjacent to fibrotic septa around the portal area, which may be explained by regional hypoxia. It is suggested that injured hepatocytes may be one of the sources of VEGF under hypoxic condition in CCl_4_-induced cirrhosis.

## Conclusions

In conclusion, YGJ and iYGJ had anti-angiogenic effects in a CCl_4_-induced cirrhosis mouse model and how this occurred is likely through improvement of hepatic hypoxia and inhibition of the HIF-1α/VEGF signaling pathway.

## Abbreviations

PDGF: Platelet derived growth factor
TGF: Transforming growth factor
FGF: Fibroblast growth factor
VEGF: Vascular endothelial growth factor
HIF: Hypoxia inducible-factor
VEGFR: Vascular endothelial growth factor reporter
YGJ: Yiguanjian
DMN: Dimethylnitrosamine
AST: Aspartate aminotransferase
ALT: Alanine aminotransferase
α-SMA: α-smooth muscle actin
CCl_4_: Carbon tetrachloride
HSC: Hepatic stellate cell
iYGJ: Ingredients of YGJ
ECM: Extracellular matrix
Hyp: Hydroxyproline
HPLC: High-performance liquid chromatography
ALB: Albumin
H&E: Hematoxylin and eosin
RT-PCR: Real time reverse transcription polymerase chain reaction

## References

[1] Bosch J, Abraldes JG, Fernández M, García-Pagán JC (2010). Hepatic endothelial dysfunction and abnormal angiogenesis: new targets in the treatment of portal hypertension. J Hepatol.

[2] Fernández M, Semela D, Bruix J, Colle I, Pinzani M, Bosch J (2009). Angiogenesis in liver disease. J Hepatol.

[3] Kajdaniuk D, Marek B, Borgiel-Marek H, Kos-Kudła B (2011). Vascular endothelial growth factor (VEGF) - part 1: in physiology and pathophysiology. Endokrynol Pol.

[4] Liu LX, Lu H, Luo Y, Date T, Belanger AJ, Vincent KA (2002). Stabilization of vascular endothelial growth factor mRNA by hypoxia-inducible factor 1. Biochem Biophys Res Commun.

[5] Lin HJ, Chen JY, Lin CF, Kao ST, Cheng JC, Chen HL (2011). Hepatoprotective effects of Yi Guan Jian, an herbal medicine, in rats with dimethylnitrosamine-induced liver fibrosis. J Ethnopharmacol.

[6] Wang XL, Jia DW, Liu HY, Yan XF, Ye TJ, Hu XD (2012). Effect of Yiguanjian decoction on cell differentiation and proliferation in CCl_4_-treated mice. World J Gastroenterol.

[7] Mu Y, Liu P, Du G, Du J, Wang G, Long A (2009). Action mechanism of Yi Guan Jian Decoction on CCl4 induced cirrhosis in rats. J Ethnopharmacol.

[8] Lin HJ, Tseng CP, Lin CF, Liao MH, Chen CM, Kao ST (2011). A Chinese herbal decoction, modified Yi Guan Jian, induces apoptosis in hepatic stellate cells through an ROS-mediated mitochondrial/caspase pathway. Evid Based Complement Alternat Med..

[9] Hu B, An HM, Shen KP, Xu L, Du Q, Deng S (2011). Modified Yi Guan Jian, a Chinese herbal formula, induces anoikis in Bel-7402 human hepatocarcinoma cells in vitro. Oncol Rep.

[10] Schnabl B, Kweon YO, Frederick JP, Wang XF, Rippe RA, Brenner DA (2001). The role of Smad3 in mediating mouse hepatic stellate cell activation. Hepatology.

[11] Zhou YN, Sun MY, Mu YP, Yang T, Ning BB, Ren S (2014). Xuefuzhuyu Decoction Inhibition of Angiogenesis Attenuates Liver Fibrosis Induced by CCl_4_ in Mice. J Ethnopharmacol.

[12] Tugues S, Fernandez-Varo G, Munoz-Luque J, Ros J, Arroyo V, Rodés J (2007). Antiangiogenic treatment with sunitinib ameliorates inflammatory infiltrate, fibrosis, and portal pressure in cirrhotic rats. Hepatology.

[13] Friedman SL (2010). Evolving challenges in hepatic fibrosis. Nat Rev Gastroenterol Hepatol.

[14] Rosmorduc O, Housset C (2010). Hypoxia: A Link between Fibrogenesis, Angiogenesis, and Carcinogenesis in Liver Disease. Semin Liver Dis.

[15] Niecknig H, Tug S, Reyes BD, Kirsch M, Fandrey J, Berchner-Pfannschmidt U (2012). Role of reactive oxygen species in the regulation of HIF-1 by prolyl hydroxylase 2 under mild hypoxia. Free Radic Res.

[16] Copple BL, Bustamante JJ, Welch TP, Kim ND, Moon JO (2009). Hypoxia-inducible factor-dependent production of profibrotic mediators by hypoxic hepatocytes. Liver Int.

[17] Yoshiji H, Kuriyama S, Yoshii J, Ikenaka Y, Noguchi R, Hicklin DJ (2003). Vascular endothelial growth factor and receptor interaction is a prerequisite for murine hepatic fibrogenesis. Gut.

[18] Zhao YZ, Wang B, Wang TC (2006). Effect of vascular endothelial growth factor in experimental biliary cirrhosis. Chin J Mod Med..

[19] Sheng CN, Luo Y, Gao W, Chen WB (2001). Relationship between vascular endothelial growth factor and hepatitis B. J Nanjing Mil Med Coll..

[20] Yan J, Chen W, Ma Y, Sun X (2000). Expression of vascular endothelial growth factor in liver tissues of hepatitis B. Zhonghua Gan Zang Bing Za Zhi.

